# Bioactive constituents, *in vitro* radical scavenging and antibacterial activities of selected *Apis mellifera* honey from Kenya

**DOI:** 10.1111/ijfs.14403

**Published:** 2019-10-25

**Authors:** Hosea O. Mokaya, Joel L. Bargul, Janet W. Irungu, Hans Michael G. Lattorff

**Affiliations:** ^1^ International Centre of Insect Physiology and Ecology (icipe) P.O. Box 30772‐00100 Nairobi Kenya; ^2^ Biochemistry Department Jomo Kenyatta University of Agriculture and Technology P.O. Box 62000‐00200 Nairobi Kenya; ^3^ German Centre for Integrative Biodiversity Research (iDiv) Halle‐Jena‐Leipzig Deutscher Platz 5e 04103 Leipzig Germany; ^4^ Naturwissenschaftliche Fakultät I Martin‐Luther‐Universität Halle‐Wittenberg 06099 Halle (Saale) Germany

**Keywords:** Bioactive phytochemicals, biofunctional properties, DPPH, flavonoids, honey, nonperoxide, phenols

## Abstract

There is limited information about the relative composition and health benefits of various honey consumed across Africa. This study aimed at estimating the bioactive constituents, *in vitro* radical scavenging and antibacterial activities of 16 kinds of honey obtained from different geographical locations in Kenya. Manuka 5 + honey was included for comparison. Some of the tested honey had biochemicals and bioactivities similar to or higher than Manuka 5 + honey. The honey exhibited DPPH radical scavenging ability, with several types of honey showing superior scavenging potential than Manuka 5 + honey, owing to their high phenol content. All types of honey inhibited the growth of *E. coli* and further showed a substantial amount of nonperoxide antimicrobial activity. The geographical origin of honey had an influence on its bioactive contents. Overall, these findings suggest that Kenyan honey has great therapeutic potential, and thus, its clinical application should not be overlooked.

## Graphical abstract

Kenya is rich in floral diversity that is unique from one geographical location to the other. This provides the honeybees an opportunity to process honey of varying composition. These honeys contain among other components, the phytochemicals like phenols, flavonoids, and other bioactives, just like manuka 5+ honey. Some of these bioactives contribute significantly to the antibacterial and antioxidant properties of honey. It can therefore be deduced that the consumption of Kenyan honey will be beneficial to human health.

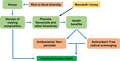

## Introduction

Honey processed by bees (*Apis mellifera L.*) from nectar, secretions of living parts of plants or excretions of plant‐sucking insects is the most utilised natural foodstuffs (Ball, 2007). The ever‐increasing awareness about honey’s nutritive value and beneficial health effects has drawn global attention resulting in its current high demand (Habib *et al.*, 2014). Honey is a mixture of mainly sugars (predominantly fructose and glucose) and water, alongside other minor constituents such as proteins, amino acids, enzymes, organic acids, vitamins, minerals, polyphenols and volatile compounds (Alvarez‐Suarez *et al.*, 2014; da Silva *et al.*, 2016). Though their concentration in honey is minor, the latter compounds have been shown to contribute significantly to honey’s biofunctional properties (Cornara *et al.*, 2017). Honey composition varies depending on its floral and geographical origin (Gambacorta *et al.*, 2014; Castiglioni *et al.*, 2018).

Currently, pathogenic microbes are proving difficult to treat due to continued evolution and emergence of drug‐resistant pathogens, for example methicillin‐resistant *Staphylococcus aureus* (MRSA), which if untreated may lead to amputation of the affected part of the body or loss of the patient (Levy & Marshall, 2004; Maddocks & Jenkins, 2013). This scenario has revitalised the search for alternative and efficient remedies, making it obligatory to re‐evaluate therapeutic uses of natural products with antimicrobial activity such as honey (Boateng & Diunase, 2015). Previous studies on honey’s antimicrobial activity showed that it enhances the healing process of infected wounds by suppressing the growth and survival of pathogenic wound‐associated microbes. This is in part due to the presence of diverse antimicrobial factors such as high osmotic pressure, high acidity, phenolic compounds, H_2_O_2_, flavonoids, antibacterial peptides, antibiotic‐like derivatives and other uncharacterised components (Al‐Waili *et al.*, 2011; Brudzynski *et al.*, 2012). The synergistic action of these components at various cellular targets reduces the ability of microbes to develop resistance to honey (Cooper *et al.,* 2010, Hussain *et al.,* 2015). For instance, Manuka honey from New Zealand displays inhibitory effects against the growth of both Gram‐positive and Gram‐negative bacteria, even those that show resistance to some antibiotics (Tan *et al.*, 2009). For these reasons, the re‐introduction of honey in our clinics as complementary medicine is well intentioned as it represents a novel remedy in combating resistant microbes.

Alongside the antimicrobial property, honey is also a natural source of antioxidants. Oxidative stress, which is a result of chemical or biological processes, has been described to be the root cause of many pathological conditions such as the ageing process, cancer, cardiovascular disease, reduced wound healing, gastrointestinal inflammatory diseases and atherosclerosis (Halliwell & Gutteridge, 2015). Natural sources of antioxidants like fruits, vegetables and honey are currently sought‐after. Honey contains bioactive compounds such as phenols, flavonoids, carotenoids, vitamins, organic acids and other compounds, which might work synergistically to provide a positive antioxidant effect (Johnston *et al.*, 2005; Gül & Pehlivan, 2018). As antioxidants, the aforementioned bioactive compounds could work through a myriad of mechanisms including donation of hydrogen atoms, scavenging for free radicals, quenching singlet oxygen, acting as substrates for some radicals and chelation of metal ions (Al‐Waili & Boni, 2003; Küçük *et al.*, 2007).

Despite Africa’s richness in floral diversity, which suggests the existence of diverse kinds of honey of assorted composition, little efforts have been made to discover new honey types with therapeutic potential. Physicochemical constituents of Kenyan *Apis mellifera* honey have been reported (Muli *et al.*, 2007; Nganga *et al.*, 2013), and further research is therefore needed to study their therapeutic potential. Consequently, the purpose of this study was to investigate the bioactive components, *in vitro* radical scavenging and antibacterial activities of 16 *Apis mellifera* honeys derived from different geographical locations in Kenya.

## Materials and methods

### Chemicals

Analytical grade chemicals of 2, 2‐diphenyl‐1‐picrylhydrazyl (DPPH), Folin–Ciocalteu’s reagent, gallic acid, quercetin, β‐carotene, bovine serum albumin (BSA), sodium carbonate (Na_2_CO_3_), aluminium chloride (AlCl_3_), sodium nitrite (NaNO_2_), catalase and sodium hydroxide (NaOH) were purchased from Sigma‐Aldrich (Kobian Kenya Ltd.). Mueller‐Hinton Agar (MHA) was purchased from Himedia Laboratories Pvt. Ltd. (F&S Scientific, Nairobi, Kenya). Chemicals of analytical grade were used in all analyses.

### Honey samples

Sixteen *Apis mellifera* honey samples were collected directly from hives maintained by local beekeepers in four different climate regions in Kenya: Kakamega (high‐rainfall), Coast (hot and wet), Mwingi (semi‐arid) and Mt. Kenya (cool and wet). From each region, four independent apiaries were selected each comprising of at least ten hives. Five hives from each apiary were randomly selected and sampled. The five honey samples were pooled to constitute one sample per apiary from the four different geographical regions (Table S1). The largely studied Manuka 5 + honey from New Zealand was purchased locally in a public super market for comparison. All samples were stored at −80 °C until fully analysed.

### Quantification of bioactive constituents

#### Total carotenoid content (TCC)

Total carotenoid content was determined by following the procedures in the published protocol (Alvarez‐Suarez *et al.*, 2010a). Briefly, 1 g of the sample was shaken in 10 mL of n‐hexane–acetone mixture (6:4) for 10 min at room temperature and filtered through Whatman No. 4 filter paper. The absorbance of the filtrate was measured at 450 nm against a blank of n‐hexane–acetone (6:4). β‐Carotene was used as standard to generate a calibration curve (0.015–0.48 µg mL^−1^). TCC was expressed as mg of β‐carotene equivalents (mg β‐carot E kg^−1^ of honey).

#### Total protein content (TP)

The total protein content was determined by the Bradford method with minor modifications (Bradford, 1976). To 0.1 mL honey solution (50% *w*/*v*), 0.9 mL of the Coomassie Brilliant Blue reagent was added. After 2 min of incubation, the absorbance was measured at 595 nm against the blank (i.e. the reactive solution without the sample) using a spectrophotometer. Bovine serum albumin was used to generate the calibration curve (0–300 μg mL^−1^) in 0.15 m sodium chloride solution. The TP content was calculated and expressed as mg of BSA/100g of honey.

#### Total phenol content (TPC)

The Folin–Ciocalteu method as described by Singleton and co‐workers (Singleton *et al.*, 1999) with minor modification was used. To 1 g of each honey sample, 20 mL of distilled water was added, and then, 1mL of the resulting solution was mixed with 5mL of 0.2 N Folin–Ciocalteu reagent for 5min. After adding 4 mL of 75 g L^−1^ sodium carbonate, the mixture was incubated at room temperature for 2 h, and then, the absorbance of the reaction mixture was read at 760 nm against water blank. Gallic acid was used as a standard to yield the calibration curve (0–250 μg mL^−1^). The total phenolic content was expressed as mg of gallic acid equivalents (mg GAE kg^−1^ of honey).

#### Total flavonoid content (TFC)

The total flavonoid content of honey samples was measured based on the aluminium chloride (AlCl_3_) colorimetric assay as previously described by Zhishen *et al. *(1999). One millilitre extract of each sample was mixed with 4 mL of distilled water before 0.3 mL of 5% NaNO_2_ was added and mixed. After 5 min, 0.3 mL of 10% AlCl_3_ was added to the mixture and left for 1 min before adding 2 mL of 1 m NaOH. Then, 2.4 mL of distilled water was added. The mixture was used to measure the absorbance against the blank at 510 nm. Quercetin was used to generate a calibration curve (20–200 μg mL^−1^), and TFC was expressed as mg quercetin equivalent per 100g of honey (mg QE/100 g honey).

### Analysis of *in vitro* radical scavenging activity

#### DPPH radical scavenging activity

DPPH assay was performed by spectrophotometry as previously reported (Chua *et al.*, 2013). To 0.75 mL of methanolic honey solution at different concentrations, 1.5 mL of DPPH solution (2 mg/100 mL methanol) was added. The mixtures were left for 15 min at room temperature in the dark, and then, the absorbances were measured at 517 nm. The blank sample consisted of 0.75 mL of honey solution with 1.5 mL of methanol, and for the control sample, 0.75 mL of methanol was mixed with 1.5 mL DPPH solution. The free radical scavenging activity was expressed as a percentage of inhibition, using the following formula:

% inhibition = [(control absorbance−sample absorbance)/control absorbance] × 100%

The concentration of honey required to inhibit 50% of the initial DPPH radical (IC_50_) was obtained from the linear regression curve, generated by data obtained from different honey concentrations between 1 and 500 mg mL^−1^, against the percentage inhibition of DPPH.

### Antimicrobial activity

#### Bacterial growth and maintenance

A few single bacterial colonies (*Escherichia coli:* ATCC 25922) from an overnight culture on Mueller‐Hinton Agar (MHA) were inoculated into sterile distilled water to achieve a turbidity of 0.5 McFarland (≈ 1 × 10^8^ CFU mL^−1^ as per Clinical and Laboratory Standards Institute) by measuring the optical density (OD) = 0.132 at 600 nm, as described in previous reports (Boateng & Diunase, 2015; Kuś *et al.*, 2016).

#### Agar well diffusion assay

This assay was performed in sterile MHA prepared in separate sterile petri dishes as previously reported, but with minor modifications (Boateng & Diunase, 2015). From overnight microbial culture (*Escherichia coli:* ATCC 25922) prepared as mentioned above, 100 μL was added to separate 100 mL sterile MHA at 45 °C, thoroughly mixed and poured into labelled sterile petri dishes. Sterile cork borer (9 mm) was used to bore two wells into each agar plate. To the wells, 100 µL of honey solutions (25% *w*/*v*, filter‐sterilised through 0.45‐μm pore filters) were added, and then, the dishes incubated for 24 h at 37 °C. Digital pictures of petri dishes with antibacterial zones of inhibition were taken, and the zone diameters were measured using the *antibiogramj* program (Alonso *et al.*, 2017). Each honey sample was assayed in duplicate.

#### Nonperoxide antimicrobial activity

Honey solutions containing catalase and bacteria were prepared in sterile distilled water to determine the nonperoxides as reported by Allen *et al. *(1991) and CO *et al. *(2013). Catalase was introduced to break down H_2_O_2_ present in honey samples and then used to generate zones of inhibition. The test contained 2.9 mL of a 25% *w*/*v* honey solution plus 0.1 mL of a 5 mg mL^−1^ catalase solution. To determine the diameters of zones of inhibition, the agar well diffusion assay was carried out as described above (2.5.2).

### Determination of physicochemical properties

The physicochemical properties were assessed following the harmonised international honey commission protocols (Bogdanov, 2009) as described in SM1.

### Data analysis

Kruskal–Wallis test (two‐tailed test) was used to compare honey samples from different locations at *P* < 0.05, with Dunn’s procedure (two‐tailed test) for multiple comparison (XLSTAT Addinsoft SARL 2019). Spearman’s rank correlation analysis was performed to evaluate the possible relationship between the studied parameters using the same software package. R working environment v3.5.0 (R Core Team 2019) along with packages *factoextra* 1.0.5 and *ggplot2* 3.1.1 was used to perform a principal components analysis.

## Results and discussions

### Bioactive compounds

#### Total carotenoid and protein contents

The results for total carotenoids content (TCC) of the investigated Kenyan honey samples are shown in Table 1. The range was between 0.32 and 3.70 mg β‐Carot E kg^−1^ of honey. These results are consistent with previously reported values by other researchers (Ferreira *et al.*, 2009; Alvarez‐Suarez *et al.*, 2010b). Some of the honeys (K2, K3, K4, C1, MK3 and MK4) had higher TCC values, thus representing an alternative natural source of carotenoids. The significant differences were observed between the mean values of honey from Kakamega and those from Coast, and Mwingi with *P *˂ 0.05 values, signifying that the quantity of carotenoids in honey depends on the distinctiveness of flora in a given geographical region. Apart from the polyphenolic contents, carotenoids also do contribute to honey colouring (Ferreira *et al.*, 2009; Alvarez‐Suarez *et al.*, 2010b).

**Table 1:** Bioactive components of Kenyan honeys (Mean ± SD)

Location	Samples	Parameters			
		Carotenoid (mg β‐Carot E kg^−1^)	Protein (mg BSA E/100 g)	TPC (mg GA E/100 g)	TFC (mg Q E/ 100 g)
Kakamega	K1	0.73 ± 0.07	27.91 ± 0.18	164.48 ± 0.69	16.58 ± 0.39
	K2	0.90 ± 0.00	40.40 ± 0.55	111.79 ± 1.65	20.36 ± 0.77
	K3	1.32 ± 0.19	37.18 ± 0.86	158.55 ± 1.19	15.02 ± 1.02
	K4	2.19 ± 0.07	41.53 ± 1.06	132.16 ± 2.30	28.13 ± 0.67
Mean		1.29 ± 0.65^a^	36.75 ± 6.18^a^	141.74 ± 24.42^a^	20.02 ± 5.85^a^
Coast	C1	1.44 ± 0.19	44.15 ± 1.65	217.63 ± 3.18	73.02 ± 3.15
	C2	0.57 ± 0.07	57.64 ± 0.70	102.11 ± 1.19	20.36 ± 0.77
	C3	0.48 ± 0.07	53.22 ± 0.66	76.90 ± 2.24	27.47 ± 3.33
	C4	0.32 ± 0.07	33.91 ± 0.53	68.05 ± 1.14	21.02 ± 1.02
Mean		0.70 ± 0.51^b^	47.23 ± 10.51^b^	116.17 ± 69.16^b^	35.47 ± 25.24^b^
Mwingi	M1	0.36 ± 0.07	47.70 ± 0.75	53.43 ± 0.42	15.47 ± 1.16
	M2	0.61 ± 0.07	60.12 ± 0.72	112.61 ± 3.62	13.47 ± 0.67
	M3	0.78 ± 0.13	34.54 ± 0.19	110.88 ± 3.16	17.69 ± 0.39
	M4	0.57 ± 0.07	33.16 ± 0.41	116.54 ± 2.47	15.91 ± 0.39
Mean		0.58 ± 0.17^b^	43.88 ± 12.65^a,b^	98.37 ± 30.05^b^	15.63 ± 1.73^c^
Mt. Kenya	MK1	0.53 ± 0.13	43.45 ± 0.42	58.09 ± 2.20	25.69 ± 0.77
	MK2	0.57 ± 0.07	40.00 ± 0.72	142.11 ± 1.19	47.02 ± 1.68
	MK3	3.70 ± 0.07	45.07 ± 1.99	32.79 ± 1.53	24.58 ± 1.39
	MK4	0.82 ± 0.07	27.43 ± 1.42	33.89 ± 2.86	19.47 ± 0.67
Mean		1.40 ± 1.54^a,b^	38.99 ± 7.99^a,b^	66.72 ± 51.60^b^	29.19 ± 12.19^b^
Control	Manuka5+	0.79 ± 0.01	46.93 ± 0.48	93.98 ± 1.41	31.22 ± 1.66

BSAE, bovine serum albumin equivalent; GAE, gallic acid equivalent; QE, quercetin equivalent; TFC, total flavonoid content; TPC, total phenolic content; β‐CarotE, β‐carotene equivalent.

The mean values for locations within a column with different letters are significantly different for *P* < 0.05 (Dunn’s test). All parameters were done in triplicate.

The mean total protein content for the analysed honey ranged from 36.75 to 47.23 mg BSA E/100 g of honey (Table 1). These results are comparable with previously reported values on honey protein content (Pérez *et al.*, 2007; Alvarez‐Suarez *et al.*, 2010b). Normally, honey protein content is less than 500 mg/100 g (Anklam, 1998). The honey protein content can be attributed to the presence of enzymes, either from the floral sources (nectar and pollen) or introduced by bees during honey processing. There was a significant difference in the mean protein content between honey from Coast and those from Kakamega (*P *˂ 0.05). Important to note is that carotenoids and proteins present in honey do not contribute much to its bioactivity, and this was ascertained by the weak correlations that were obtained between TCC and antiradical activity, expressed as 1/IC_50_ (*r*_s_ = 0.29, *P *> 0.05); TCC and antibacterial activity (*r*_s_ = 0.00, *P* > 0.05); protein and antiradical activity, expressed as 1/IC_50_ (*r*_s_ = 0.18, *P* > 0.05); and between protein and antibacterial activity (*r*_s _= −0.26, *P* > 0.05), as stated in Table 3.

#### Total phenols and flavonoid content

The mean total phenols content (TPC) of Kenyan honey varied between 66.72 and 141.74 mg GA E/100 g of honey (Table 1). A similar level of TPC was observed in past studies (Ouchemoukh *et al.*, 2007; Attanzio *et al.*, 2016; Boussaid *et al.*, 2018). Generally, the concentration of phenols in honey varies depending on its botanical and geographical origin (Küçük *et al.*, 2007). The average TPC of different regions varied significantly for Kakamega and Mwingi, Kakamega and Coast, and Kakamega and Mt. Kenya with *P *˂ 0.05 values. The phenols have been shown to be the main contributors to the health‐promoting properties of honey (Alvarez‐Suarez *et al.*, 2010b; Chua *et al.*, 2013). This was in agreement with the positive correlations that were found between the TPC and radical scavenging activity (*r*_s_ = 0.72, *P *˂ 0.05), and TPC and antibacterial activity (*r*_s_ = 0.73, *P *˂ 0.05) as indicated in Table 3. A previous study also reported that the antibacterial activity of honey is linked with its phenol content (Silici *et al.*, 2010). Thus, most of the studied local honey could be considered to have more health benefits, due to their high phenol content.

The mean total flavonoid content (TFC) is shown in Table 1. The values ranged from 13.47 to 73.02 mg Q E/100 g of honey. These values were slightly higher than the values reported in previous studies (Meda *et al.*, 2005; Liberato *et al.*, 2011) but were within the range stated by Habib and colleagues (Habib *et al.*, 2014). The means between Kakamega and Coast, Kakamega and Mwingi, and Kakamega and Mt. Kenya were significantly different with *P *˂ 0.05 values, while the means between Coast and Mwingi, and Mwingi and Mt. Kenya were significantly different with *P *˂ 0.0001 values. The comparison of Coast and Mt. Kenya had no significant difference (*P* > 0.05). This indicates that honey’s flavonoid content greatly depends on the distinctiveness of the flora in a given geographical area.

### DPPH radical scavenging activity

#### DPPH radical scavenging activity expressed as IC_50_

**Figure 1:**
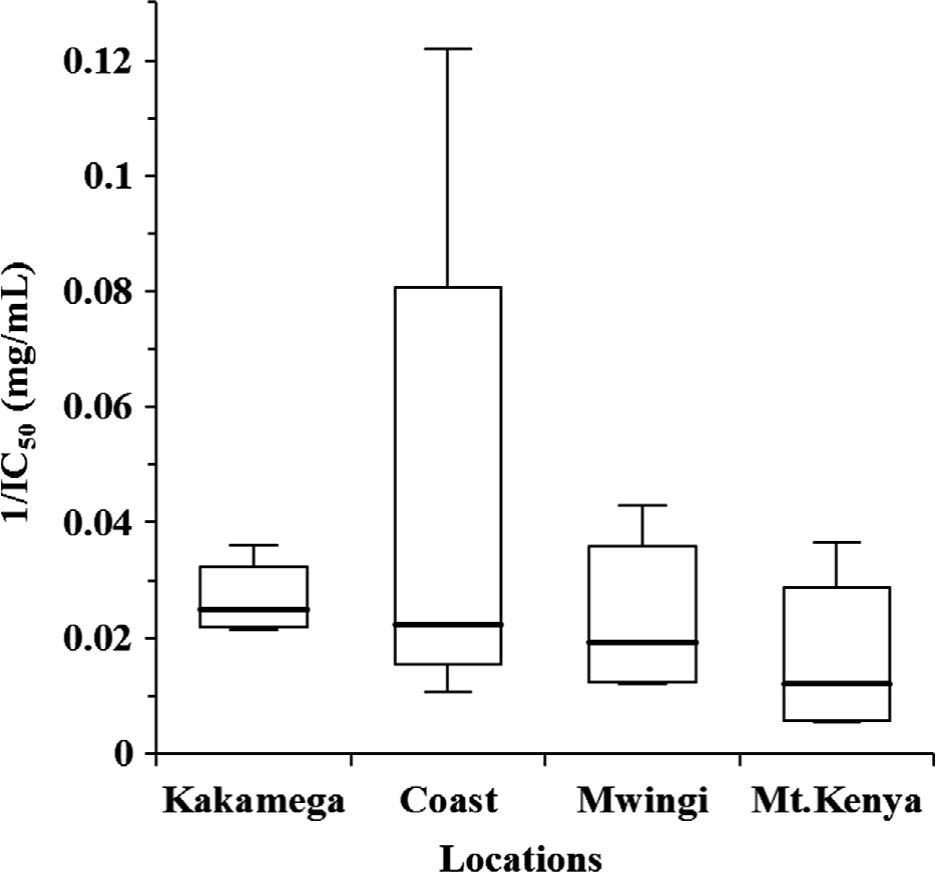
Box plot diagram of antiradical activity showing the differences among geographical locations of honey sampling. The horizontal line represents the median, the box shows the 25th and 75th percentiles, and the whiskers represent the 5th and 95th percentiles.

A lower IC_50_ value (mg mL^−1^) indicates a greater ability of the sample to neutralise the free radical. The IC_50_ values ranged from 186.85 mg mL^−1^ (being the least effective) to 8.20 mg mL^−1^ (being the most effective) as shown in Table S2. In previous studies, IC_50_ values ranged from 4.2 to 106.72 mg mL^−1^ (Liberato *et al.*, 2011), 12.56 to 152.40 mg mL^−1^ (Can *et al.*, 2015) and 25.45 to 294.26 mg mL^−1^ (do Nascimento *et al.*, 2018). Therefore, it can be inferred that the tested Kenyan honey had DPPH radical scavenging potential comparable with those reported by other scholars. In the present study, some of the Kenyan honey (K3, C1, M3 and MK2) had higher DPPH radical scavenging activity, with IC_50_ values lower than 30 mg mL^−1^ (Table S2). Regional comparison showed that the means for honey from Kakamega and Mt. Kenya (*P *˂ 0.05) differed significantly (Table S2). Overall, honey from Kakamega had the highest radical scavenging activity (Fig. 1). Positive correlation was recorded between DPPH radical scavenging activity and TPC (*r*_s_ = 0.72, *P *˂ 0.05) as defined in Table 3. These results are in perfect accordance with the reports of other authors (Alvarez‐Suarez *et al.*, 2010b; Sant’Ana *et al.*, 2012; Boussaid *et al.*, 2018).

### Antimicrobial activity

#### Agar well diffusion assay

All the tested honeys exhibited antibacterial activity as shown in Table 2. From these results, it is evident that all the investigated honey had a more potent antibacterial activity against *E. coli*, as demonstrated by larger inhibition zones. These results concurred with the findings of previous studies where this strain was reported to be the most susceptible (Zainol *et al.*, 2013; Boateng & Diunase, 2015). The means of the data grouped by sampling locations are not significantly different.

**Table 2:** Antibacterial activities of Kenyan honeys against *E. coli* ATCC 25922 (Mean ± SD in mm)

Location	Samples	*E. coli* (ATCC 25922)	
		Whole activity	Nonperoxide
Kakamega	K1	33.3 ± 0.35	30.0 ± 0.71
	K2	31.3 ± 1.06	27.3 ± 1.06
	K3	31.8 ± 0.35	24.3 ± 0.35
	K4	30.3 ± 0.35	28.0 ± 0.00
Mean		31.6 ± 1.25	27.4 ± 2.36
Coast	C1	34.3 ± 0.35	29.3 ± 0.35
	C2	30.5 ± 0.71	29.3 ± 0.35
	C3	30.3 ± 0.35	25.8 ± 0.35
	C4	30.5 ± 0.71	27.5 ± 1.41
Mean		31.4 ± 1.92	27.9 ± 1.68
Mwingi	M1	30.3 ± 0.35	26.5 ± 0.71
	M2	30.3 ± 0.35	26.5 ± 0.71
	M3	30.5 ± 0.00	26.5 ± 0.71
	M4	32.8 ± 0.35	27.8 ± 1.06
Mean		30.9 ± 1.21	26.8 ± 0.65
Mt. Kenya	MK1	31.0 ± 0.71	27.8 ± 0.35
	MK2	33.3 ± 0.35	29.8 ± 0.35
	MK3	29.3 ± 0.35	25.8 ± 0.35
	MK4	29.0 ± 0.00	27.3 ± 0.35
Mean		30.6 ± 1.96	27.6 ± 1.65
Control	Manuka 5+	32.3 ± 0.35	27.5 ± 0.71

There were no significant differences between sampling locations for *P* < 0.05 (Dunn’s test). Each assay was done in duplicate.

**Figure 2:**
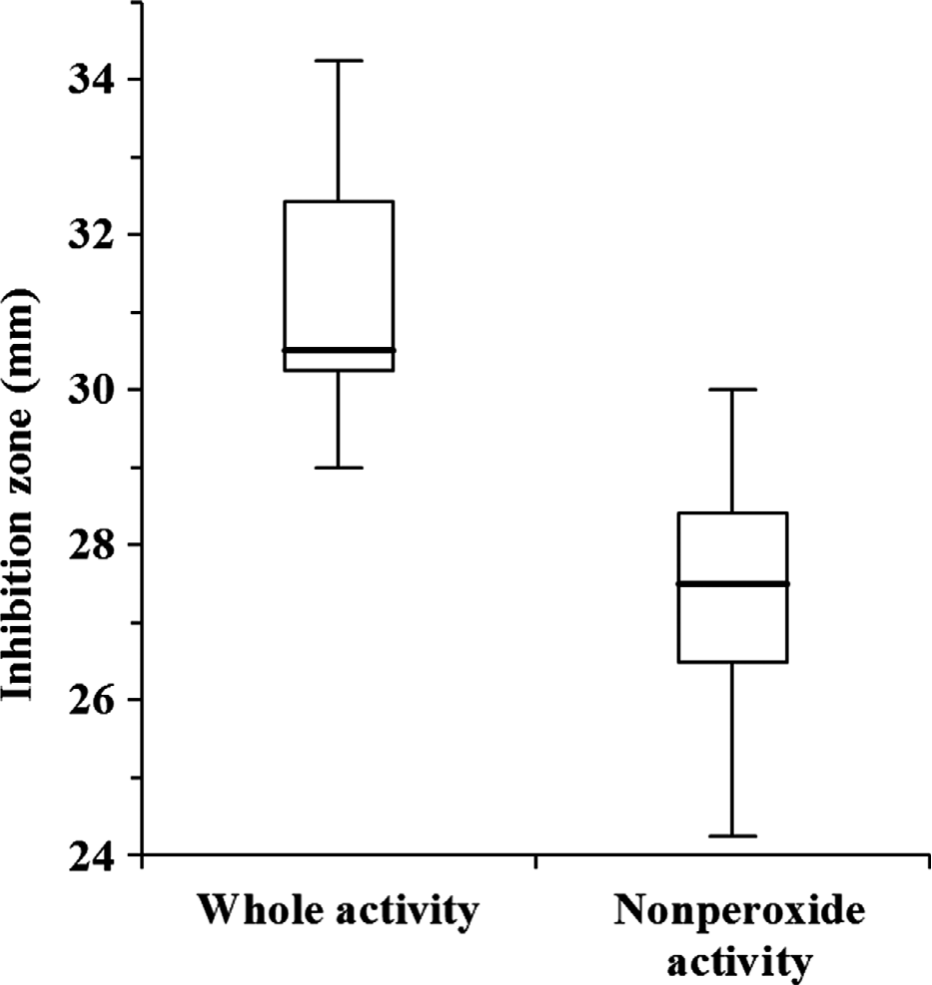
Box plot diagram of antibacterial activity. The horizontal line represents the median, the box shows the 25th and 75th percentiles, and the whiskers represent the 5th and 95th percentiles. The two bioassays were significantly different for *P* < 0.05 (Kruskal–Wallis test).

From the findings, all the tested Kenyan honey had a high nonperoxide activity similar to Manuka 5 + honey. There was substantial variability both within and among regions (Table 2). The antibacterial activity of the honeys was not affected by the absence of H_2_O_2_, but still important to note is that H_2_O_2_ remains to be one of the major components of honey’s antibacterial property, as there was a significant difference (*P *˂ 0.0001) between the nonperoxide and whole activity assays (Fig. 2). This was further corroborated by the calculated high percentage of H_2_O_2_ contribution values (Table S2). A previous study also showed that honey treated with catalase had higher MIC values (reduced activity), an indication that H_2_O_2_ exerts a significant antibacterial activity (Stagos *et al.*, 2018). Apart from H_2_O_2_, the antibacterial property of honey has been attributed to other factors such as high osmotic pressure, high acidity, phenolic compounds, flavonoids, antibacterial peptides, antibiotic‐like derivatives and other uncharacterised components (Al‐Waili *et al.*, 2011; Brudzynski *et al.*, 2012). These analyses revealed strong positive correlations between the antibacterial activity and TPC (*r*_s_ = 0.73, *P *˂ 0.05) and between the antibacterial whole activity and nonperoxide (*r*_s_ = 0.58, *P *˂ 0.05) as specified in Table 3. However, there were no significant differences between the mean values of honey as grouped by sampling locations.

**Table 3:** Correlation matrix of the studied parameters based on Spearman’s rank correlation coefficients (*r*_s_)

	TPC	TFC	Protein	Carotenoid	1/IC_50_	Antibacterial (whole)	Antibacterial (nonperoxide)	H_2_O_2_ contribution (%)
TPC	–							
TFC	0.02	–						
Protein	−0.20	0.16	–					
Carotenoid	0.28	0.16	−0.11	–				
1/IC_50_	0.72*	0.26	0.18	0.29	–			
Antibacterial (whole)	0.73*	0.22	−0.26	0.00	0.47	–		
Antibacterial (nonperoxide)	0.46	0.41	−0.22	−0.09	0.13	0.58**	–	
H_2_O_2_ contribution (%)	0.25	−0.11	0.11	0.07	0.39	0.33	−0.48	–

* *P* ˂ 0.01.

** *P *˂ 0.05.

### Principal Component Analysis (PCA)

**Figure 3:**
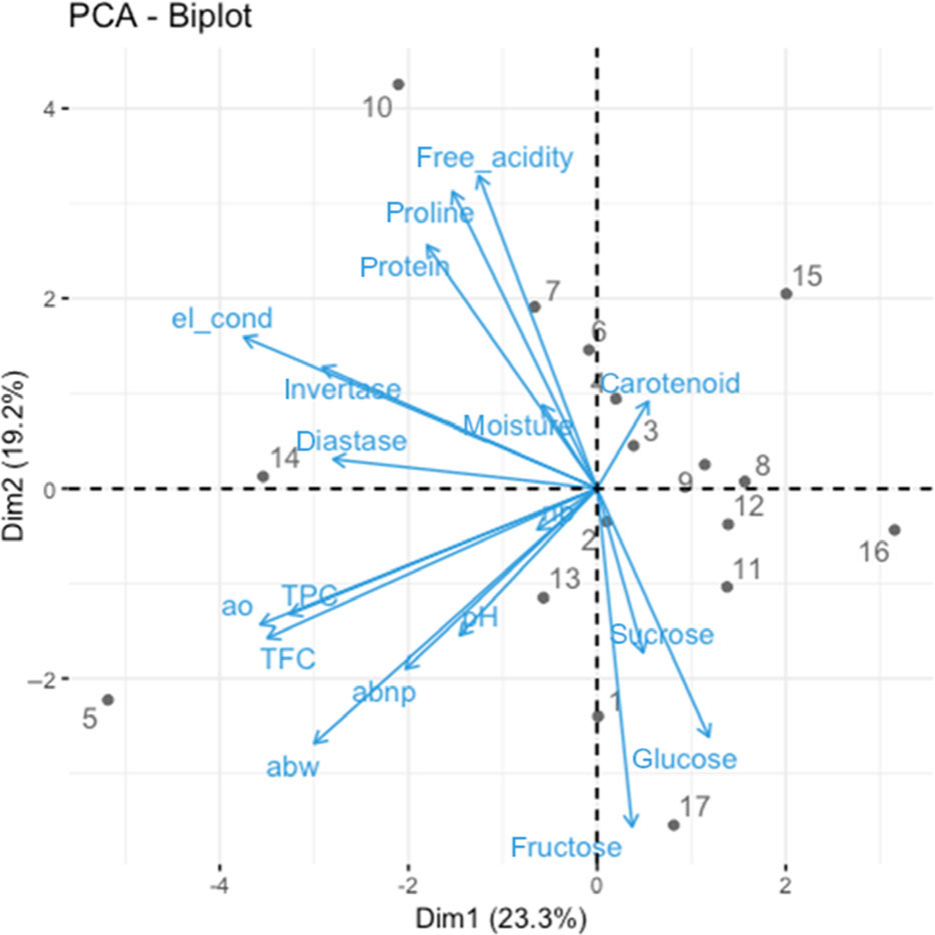
Biplot of the first two components obtained by PCA performed with data obtained from all assayed parameters. Arrows indicate the eigenvectors for assayed parameters, and dots represent honey samples (1–4 = Kakamega, 5–8 = Coast, 9–12 = Mwingi, 13–16 Mt. Kenya, 17 = Manuka5+). abnp, antibacterial (nonperoxide activity); abw, antibacterial (whole activity); ao, antioxidant (1/IC_50_); el_cond, electrical conductivity; nb, H_2_O_2_ contribution; TFC, total flavonoid content; TPC, total phenolic content. [Colour figure can be viewed at wileyonlinelibrary.com]

PCA is one of the analytical approaches that allows the classification of complex conditions by mining information from multivariate experimental data. All assayed parameters, that is physicochemical (Table S3), bioactive and biofunctional properties, were analysed using PCA to investigate similarities among the samples and assayed variables. PCA results are presented in Fig. 3. PC1 and PC2 together explained 42.5% of the total variance. It was evident that the data obtained from the assayed parameters were not able to fully categorise our honey samples into sampling locations. However, some geographic clustering could be observed, for example samples 2 (K2), 3 (K3) and 4 (K4) from Kakamega, and samples 9 (M1), 11 (M3) and 12 (M4) from Mwingi, along the first component PC1 on the positive side. Samples from Coast and Mt. Kenya show high levels of variability due to more heterogeneous composition of honey. PC1 is strongly influenced by the bioactive properties and by the presence of enzymes, while PC2 is more strongly influenced by sugar content and protein/amino acid content.

The second component PC2 separated the parameters into two major groups: those that have a minor (diastase, invertase, protein, proline, conductivity, moisture, acidity and carotenoids) and higher (pH, TPC, TFC, sucrose, glucose and fructose) contribution to the biofunctional properties (antioxidant and antibacterial) of honey to the positive and negative sides, respectively. It was also clear from the PCA‐Biplot that TPC, TFC and antioxidant activity had a strong association, whereas pH closely interacted with whole and nonperoxide antibacterial activity.

## Conclusion

The findings of the present study allow us to understand that Kenyan *A. mellifera* honey has bioactive contents and biofunctional properties in the range or higher than other honey reported in the literature. Furthermore, this is the first study to show that some Kenyan honey has biochemicals and bioactivities comparable to the popularly studied Manuka 5 + honey.

The potential health benefits of Kenyan honey should not be overlooked, but further studies should focus on testing the sensitivity of a wide range of pathogenic microbes, quantifying the individual phenols, as these compounds considerably contributed to the honey samples’ biological properties.

Honey shows some characteristics related to the geographical origin potentially linked to differences in the vegetation and availability of melliferous plants. However, variance within geographic regions is still high and does not allow to separate honey according to regions. Hence, botanical origin of honey is still an essential factor that needs further investigation.

## Conflicts of interest

The authors declare no conflict of interest.

## Ethical guidelines

Ethics approval was not required for this research.

## Data availability statement

The data that support the findings of this study are available from the corresponding author upon reasonable request.

## Supporting information


**Table S1.** Sampling sites and GPS coordinates.
**Table S2.** DPPH radical scavenging activity (IC_50_) and % H_2_O_2_ contribution (mean ± SD).
**Table S3.** Physicochemical properties of Kenyan and manuka 5+ honey.
