# Supplementary material for: Bioactive constituents, in vitro radical scavenging and antibacterial activities of selected Apis mellifera honey from Kenya
Source: Int J Food Sci Technol. 2019 Oct 25;55(3):1246–54. doi: 10.1111/ijfs.14403 (PMC7540667; doi:10.1111/ijfs.14403)
Supplement: Supplementary file 1 — Table S1. Sampling sites and GPS coordinates. Table S2. DPPH radical scavenging activity (IC50) and % H2O2 contribution (mean ± SD). Table S3. Physicochemical properties of Kenyan and manuka 5+ honey. [file IJFS-55-1246-s001.docx]

**Appendix S1**

**Supplementary material 1 (SM1)**

**2.6 Physicochemical analysis**

Physicochemical parameters were determined as per the International Honey Commission procedures. (Bogdanov *et. al,* 2009).

**2.6.1 pH and free acidity**

A pH meter (Jenway Model 3540, Essex, England) was used to measure the pH of a 10g honey in 75mL of carbon dioxide free distilled water. Determination of free acidity was done by titrating the same sample solution with 0.1M NaOH until pH 8.3.

**2.6.2 Moisture content**

The moisture content was determined using a refractometric method. The refractive indices of honey samples were measured at ambient temperature using an Atago handheld refractometer and the corresponding moisture contents (%) were then calculated.

**2.6.3 Electrical conductivity**

Electrical conductivity was measured using a conductivity meter (3540, Jenway) for a 10% (w/v) solution of honey suspended in distilled water. The meter was calibrated with conductivity solution before taking the readings of honey solutions.

**2.6.4 Diastase**

The spectrophotometrical method was used to assess the diastase activity using a 6850 UV/ Vis spectrophotometer (Jenway). Diastase activity was determined using a buffered solution of soluble starch and honey incubated in a thermostatic bath at 40°C. The diastase value was calculated using the time taken for the absorbance to reach 0.235 at 660nm, and the results were expressed in Gothe degrees as the amount (mL) of 1% starch hydrolyzed by an enzyme in 1g of honey in 1h.

Diastase in Sunits = 300/t_x_

Where t_x_ = time taken for the absorbance to reach 0.235

**2.6.5 Invertase**

Invertase activity was determined spectrophotometrically using a 6850 UV/ Vis spectrophotometer (Jenway). 5g of honey sample were dissolved in a buffer solution (prepared by dissolving 11.66g of potassium hydrogen phosphate and 2.56g of disodium hydrogen phosphate in water and diluting to 1L), quantitatively transferred to a 25ml flask and made up to the mark with the same solution. 5ml of the substrate solution (prepared by dissolving 6.0252g of p-nitrophenyl –α-D- glucopyranoside in buffer solution and made up to 1L) was placed in two separate test tubes and incubated in a water bath at 40^o^C for 5min. Thereafter, 0.5ml of the honey solution was added to one test tube (sample) and to the other test tube (reference) 0.5ml of reaction –terminating solution (prepared by dissolving 363.42g of tris- (hydroxymethyl) amino methane in water and diluted to 1L), was added, mixed well, followed by addition of 0.5ml of honey solution. The sample solution was incubated further for 20min before 0.5ml of reaction –terminating solution was added and mixed thoroughly. The two solutions were cooled rapidly to room temperature after which the absorbances of the sample solutions and the reference were measured in 4ml cuvettes at 400nm. The reading were taken after 15min and within 1hr.

Invertase in invertase number (IN) = 21.64 x ∆ A_400 nm_

Where: 21.64 is the slope

**2.6.6 Proline content**

The spectrophotometrical method was used to determine proline content using a 6850 UV/ Vis spectrophotometer (Jenway). 0.5mL of the honey solution (5% w/v) was placed in one tube, 0.5mL of water (blank test) into a second tube and 0.5mL of proline standard solution into two other tubes. 1mL of formic acid was added followed by addition of 1ml of ninhydrin solution (3% w/v in ethylene glycol monomethyl ether) to each tube. The tubes were carefully capped and shaken vigorously for 15min before transferring them into a boiling water bath for 15min, immersing both tubes below the level of the solution. Thereafter, the tubes were moved to a water bath at 70^o^C for 10min. Finally, 5mL of the 2-propanol-water-solution was added to each tube, capped immediately then left to cool before the absorbances were measured 45min after removing from the 70^o^C water bath at the maximum near 510nm, using 4mL cuvettes.

Proline content in mg/kg honey was calculated according to the following equation: $Proline content \left( \mathrm{mg}/\mathrm{kg} \right)= \frac{\mathrm{Es}}{\mathrm{Ea}}* \frac{E1}{E2}*80$

Where;

E = Absorbance of the sample solution

Ea = Absorbance of the proline standard solution (average of two readings),

E1 = mg proline taken for the standard solution preparation

E2 = Weight of honey in grams.

80 Dilution factor

**2.6.7 Sugars**

Analysis of sugars (glucose, fructose, sucrose and maltose) was performed by High Pressure Liquid Chromatography. The chromatographic mobile phase consisted of a mixture of water: acetonitrile (25:75, v/v), whose flow was kept constant at 1mL min^−1^. The HPLC equipment comprised a binary pump, an auto sampler, and a refractive index detector, all from Agilent Technologies. Separation was performed on a 5μm LC-NH_2_ column (Supelco, Bellefonte, PA, USA) of 250mm × 4.6mm.

The external calibration curves produced by standard solutions were used to quantify the amount of sugars in the samples and the results were expressed as gram sugar per 100 g of honey.

Table S1: Sampling sites and GPS coordinates

| Location | Samples | GPS coordinates | |
| --- | --- | --- | --- |
|  |  | **Latitude** | **Longitude** |
| Kakamega (high rainfall) | K1 | 0°15'27.7"N | 34°45'03.9"E |
|  | K2 | 0°17'40.9"N | 34°30'54.9"E |
|  | K3 | 0°15'43.0"N | 34°55'40.3"E |
|  | K4 | 0°16'02.3"N | 34°27'44.4"E |
| Coast (hot and wet) | C1 | 3°32'51.6"S | 39°25'38.0"E |
|  | C2 | 3°53'45.5"S | 39°36'28.0"E |
|  | C3 | 3°10'51.9"S | 40°00'40.3"E |
|  | C4 | 4°33'20.4"S | 39°07'42.6"E |
| Mwingi (semi-arid) | M1 | 1°08'01.3"S | 37°25'12.1"E |
|  | M2 | 0°29'36.2"S | 38°11'42.4"E |
|  | M3 | 0°27'41.1"S | 38°05'05.8"E |
|  | M4 | 0°59'29.9"S | 38°28'20.2"E |
| Mt. Kenya (cool and wet) | MK1 | 0°00'31.9"N | 37°20'55.1"E |
|  | MK2 | 0°02'33.2"N | 37°16'39.0"E |
|  | MK3 | 0°06'06.5"N | 37°10'14.5"E |
|  | MK4 | 0°00'01.5"N | 37°19'45.2"E |

Table S2: DPPH radical scavenging activity (IC_50_) and % H_2_O_2_ contribution (mean ± SD)

| Location | Samples | IC_50_ (mg/mL) | H_2_O_2_ contribution (%) |
| --- | --- | --- | --- |
| Kakamega | K1 | 46.6 ± 0.33 | 9.8 ± 2.15 |
|  | K2 | 44.3 ± 0.42 | 12.8 ± 0.30 |
|  | K3 | 27.8 ± 0.59 | 23.6 ± 0.20 |
|  | K4 | 36.9 ± 0.37 | 7.4 ± 0.75 |
| Mean |  | 38.9 ± 8.50^a^ | 13.4 ± 6.19^a, b^ |
| Coast | C1 | 8.2 ± 0.08 | 14.6 ± 1.35 |
|  | C2 | 45.7 ± 1.46 | 4.1 ± 2.40 |
|  | C3 | 43.9 ± 0.84 | 14.9± 1.55 |
|  | C4 | 93.3 ± 1.39 | 9.8 ± 1.80 |
| Mean |  | 47.77 ± 34.94^a, b^ | 10.9 ± 4.36^a, b^ |
| Mwingi | M1 | 82.1 ± 3.72 | 12.4 ± 2.40 |
|  | M2 | 38.87±0.56 | 12.4 ± 0.90 |
|  | M3 | 23.3 ± 0.64 | 13.1 ± 1.65 |
|  | M4 | 79.3 ± 0.49 | 15.2 ± 1.65 |
| Mean |  | 55.89 ± 29.36^a, b^ | 13.3 ± 1.17^b^ |
| Mt. Kenya | MK1 | 55.3 ± 2.05 | 10.5 ± 0.65 |
|  | MK2 | 27.4 ± 0.44 | 10.5 ± 0.10 |
|  | MK3 | 168.1 ± 10.45 | 12.0 ± 0.10 |
|  | MK4 | 186.85 ± 11.23 | 6.0 ± 0.85 |
| Mean |  | 109.4 ± 79.797^b^ | 9.7 ± 2.81^a^ |
| Control | Manuka 5+ | 40.8 ± 0.45 | 14.7 ± 2.20 |

The mean values for locations within a column with different letters are significantly different for P < 0.05 (Dunn’s test).

Table S3: Physicochemical properties of Kenyan and manuka 5+ honey

| **Location** | **Samples** | **Moisture (%)** | **pH** | **Free acidity (meq/kg)** | **Electrical conductivity (S/cm)** | **Diastase (S units** | **Invertase (IN)** | **Proline (mg/kg)** | **Fructose (%)** | **Glucose (%)** | **Sucrose (%)** |
| --- | --- | --- | --- | --- | --- | --- | --- | --- | --- | --- | --- |
| **Kakamega** | K1 | 20.3±0.00 | 4.1±0.00 | 35.2±0.24 | 0.4±0.00 | 26.6±0.51 | 9.9±1.75 | 441.3±5.78 | 38.2±0.81 | 32.9±1.01 | 0.0±0.00 |
|  | K2 | 19.7±0.00 | 4.2±0.01 | 43.5±2.12 | 0.5±0.01 | 23.8±0.43 | 15.9±0.37 | 678.2±19.60 | 38.6±0.04 | 30.6±1.90 | 0.0±0.00 |
|  | K3 | 21.9±0.00 | 3.6±0.01 | 55.2±0.24 | 0.4±0.00 | 22.6±0.36 | 14.3±0.52 | 665.3±6.54 | 34.6±1.06 | 28.1±3.04 | 1.1±1.12 |
|  | K4 | 19.8±0.00 | 3.7±0.01 | 76.8±2.25 | 0.4±0.00 | 19.5±0.47 | 19.1±0.22 | 641.4±11.51 | 35.5±0.45 | 30.4±0.52 | 0.0±0.00 |
| **Mean** | | 20.4±0.88^a^ | 3.9±0.25 | 52.7±15.66^a, b^ | 0.4±0.06 | 23.1±2.54 | 14.8±3.32^a^ | 606.5±96.30 | 36.7±1.71 | 30.5±1.68^a, b^ | 0.3±0.48 |
| **Coast** | C1 | 21.0±0.00 | 4.1±0.03 | 35.3±0.24 | 0.8±0.01 | 23.7±1.21 | 32.1±0.24 | 500.2±11.36 | 37.0±0.97 | 29.1±0.03 | 0.0±0.00 |
|  | C2 | 20.6±0.00 | 3.7±0.01 | 71.7±2.36 | 0.4±0.00 | 22.2±0.85 | 23.8±0.09 | 614.1±35.69 | 34.3±2.00 | 29.9±1.26 | 0.0±0.00 |
|  | C3 | 22.7±0.05 | 4.3±0.00 | 51.7±2.36 | 0.7±0.00 | 22.3±0.96 | 28.1±1.36 | 698.8±7.41 | 31.7±0.52 | 27.0±1.94 | 0.3±0.12 |
|  | C4 | 23.0±0.05 | 3.9±0.02 | 55.0±0.41 | 0.3±0.00 | 20.0±1.26 | 5.8±0.01 | 737.7±1.16 | 37.2±0.08 | 33.2±1.12 | 0.0±0.00 |
| **Mean** | | 21.8±1.06^b^ | 4.0±0.22 | 53.4±12.90^a^ | 0.5±0.19 | 22.0±1.33 | 22.5±10.06^b^ | 637.7±91.10 | 35.1±2.25 | 29.8±2.23^a^ | 0.1±0.13 |
| **Mwingi** | M1 | 19.6±0.05 | 4.4±0.01 | 40.2±0.24 | 0.4±0.00 | 17.5±0.62 | 22.4±2.75 | 768.0±95.09 | 38.4±0.47 | 31.9±1.92 | 0.0±0.00 |
|  | M2 | 19.7±0.05 | 4.0±0.02 | 121.7±2.36 | 1.1±0.00 | 33.3±0.22 | 18.8±1.33 | 1394.9±23.21 | 33.2±0.84 | 29.9±1.24 | 0.0±0.00 |
|  | M3 | 21.0±0.00 | 3.8±0.02 | 50.7±0.24 | 0.2±0.00 | 24.2±1.40 | 9.0±0.00 | 591.7±1.13 | 41.1±0.35 | 35.8±0.50 | 0.0±0.00 |
|  | M4 | 20.1±0.14 | 3.7±0.00 | 50.0±0.00 | 0.3±0.00 | 8.5±0.41 | 4.5±0.75 | 503.4±17.24 | 35.3±0.40 | 26.1±1.75 | 0.0±0.00 |
| **Mean** | | 20.1±0.57^a^ | 3.9±0.26 | 65.6±32.62^a^ | 0.5±0.36 | 20.9±9.09 | 13.7±7.24^a, b^ | 814.5±348.38 | 37.0±2.98 | 30.9±3.51^a, b^ | 0.0±0.00 |
| **Mt. Kenya** | MK1 | 18.1±0.05 | 4.5±0.01 | 40.7±0.24 | 0.5±0.00 | 42.8±1.11 | 34.2±0.01 | 582.5±1.91 | 42.3±0.80 | 36.1±1.03 | 0.0±0.00 |
|  | MK2 | 20.5±0.00 | 4.0±0.01 | 53.5±2.12 | 0.6±0.00 | 46.0±1.83 | 37.6±2.03 | 1185.7±54.51 | 35.5±0.29 | 31.5±0.80 | 0.0±0.00 |
|  | MK3 | 19.4±0.17 | 3.8±0.02 | 40.2±0.24 | 0.3±0.00 | 17.5±0.96 | 22.3±0.57 | 874.4±14.47 | 33.0±1.77 | 30.2±2.76 | 0.0±0.00 |
|  | MK4 | 19.2±0.05 | 3.8±0.02 | 21.8±2.25 | 0.1±0.00 | 16.4±0.40 | 16.7±0.12 | 523.3±33.64 | 34.3±0.76 | 34.3±0.36 | 0.0±.0.00 |
| **Mean** | | 19.3±0.84^c^ | 4.0±0.28 | 39.0±11.28^b^ | 0.4±0.19 | 30.7±13.79 | 27.7±8.50^c^ | 791.5±263.59 | 36.3±3.62 | 33.0±2.29^b^ | 0.0±0.00 |
| **Control** | Manuka 5+ | 18.7±0.08 | 4.5±0.07 | 41.7±2.36 | 0.6±0.04 | 18.3±0.46 | 1.3±0.12 | 627.9±17.77 | 49.0±1.74 | 39.8±1.10 | 2.0±0.19 |

The mean values for locations within a column with different letters are significantly different for P < 0.05 (Dunn’s test).
